# WISP-3 inhibition of miR-452 promotes VEGF-A expression in chondrosarcoma cells and induces endothelial progenitor cells angiogenesis

**DOI:** 10.18632/oncotarget.17142

**Published:** 2017-04-17

**Authors:** Chih-Yang Lin, Huey-En Tzeng, Te-Mao Li, Hsien-Te Chen, Yi Lee, Yi-Chen Yang, Shih-Wei Wang, Wei-Hung Yang, Chih-Hsin Tang

**Affiliations:** ^1^ Graduate Institute of Basic Medical Science, China Medical University, Taichung, Taiwan; ^2^ Graduate Institute of Cancer Biology and Drug Discovery, College of Medical Science and Technology, Taipei Medical University, Taipei, Taiwan; ^3^ Department of Internal Medicine, Division of Hematology and Oncology, Taipei Medical University Hospital, Taipei, Taiwan; ^4^ School of Chinese Medicine, China Medical University, Taichung, Taiwan; ^5^ Department of Orthopedic Surgery, China Medical University Hospital, Taichung, Taiwan; ^6^ School of Pharmacy, China Medical University, Taichung, Taiwan; ^7^ Department of Nursing, National Taichung University of Science and Technology, Taichung, Taiwan; ^8^ Department of Medicine, Mackay Medical College, New Taipei City, Taiwan; ^9^ Department of Orthopedic Surgery, Taichung Hospital, Ministry of Health and Welfare, Taichung, Taiwan; ^10^ Department of Pharmacology, School of Medicine, China Medical University, Taichung, Taiwan; ^11^ Department of Biotechnology, College of Health Science, Asia University, Taichung, Taiwan

**Keywords:** WISP-3, VEGF-A, angiogenesis, miR-452

## Abstract

Chondrosarcoma is the second most prevalent general primary tumor of bone following osteosarcoma. Chondrosarcoma development may be linked to angiogenesis, which is principally elicited by vascular endothelial growth factor-A (VEGF-A). VEGF-A level has been recognized as a prognostic marker in angiogenesis. WNT1-inducible signaling pathway protein-3 (WISP)-3/CCN6 belongs to the CCN family and is involved in regulating several cellular functions, including cell proliferation, differentiation, and migration. Nevertheless, the effect of WISP-3 on VEGF-A production and angiogenesis in human chondrosarcoma remains largely unknown. This current study shows that WISP-3 promoted VEGF-A production and induced angiogenesis of human endothelial progenitor cells. Moreover, WISP-3-enhanced VEGF-A expression and angiogenesis involved the c-Src and p38 signaling pathways, while miR-452 expression was negatively affected by WISP-3 via the c-Src and p38 pathways. Our results illustrate the clinical significance of WISP-3, VEGF-A and miR-452 in human chondrosarcoma patients. WISP-3 may illustrate a novel therapeutic target in the metastasis and angiogenesis of chondrosarcoma.

## INTRODUCTION

Chondrosarcoma is the second most prevalent (or frequent) general primary tumor of bone, occurring predominantly in adults aged over 40 years [1]. Research groups have identified chondrosarcoma to be a metastatic and pathologically diverse malignant cancer with poor disease development [2, 3]. At present the only available treatment is surgical resection, due to limited response to radiotherapy and chemotherapy. However, recurrence is common after surgical resection, due to the high potential of this disease for metastatic propensity. There is an urgent need for a novel targeted treatment to treat chondrosarcoma metastasis [4].

Accumulating evidence has concentrated on the effect of angiogenesis in tumor proliferation, migration, and metastasis [5–7]; tumor angiogenesis occurs as a result of a shift in the balance between proangiogenic and anti-angiogenic factors [8]. Vascular endothelial growth factor-A (VEGF-A) is a key regulator of angiogenesis [9, 10]. We have previously indicated that VEGF-A expression is associated with the clinical stages of chondrosarcoma [11]. It is therefore important to examine the mechanism of VEGF-A expression in human chondrosarcoma.

MicroRNAs (miRNAs) are small non-coding RNAs that inhibit translation or affect the stability of target transcripts [7, 12]. miRNAs bind to the 3′-untranslated region (3′-UTR) of cognate messenger RNAs (mRNAs) through fully complementary or imperfect base-pairing and thus repress the translation or decrease the stability of the bound mRNAs [13, 14]. miRNAs are involved in biologic and pathologic processes including cell differentiation, proliferation, autophagy, apoptosis, migration, metastasis, and angiogenesis [15]. Numerous reports indicate that miRNAs suppress tumor dissemination and angiogenesis by inhibiting miR/VEGF-A signaling [16, 17]. For example, miR-199a has been shown to reduce angiogenesis in chondrosarcoma cells by directly inhibiting VEGF-A production [18], while miR-206 diminishes the production of VEGF-A, subsequently reducing cancer angiogenesis in renal carcinoma [19]. It is therefore necessary to identify the mechanisms underlying VEGF-A-regulated angiogenesis in order to discover novel therapeutic strategies that could be used in cancer.

Increasing reports have shown that the CCN (Cyr61, CTGF, and Nov) membrane proteins play important roles in tumorigenesis [20]. WNT1-inducible signaling pathway protein-3 (WISP)-3/CCN6 belongs to the CCN family and is involved in the regulation of many developmental effects [21]. It has been suggested that WISP-3 is downregulated in highly aggressive forms of breast cancer [22]. However, we have previously indicated that WISP-3 promotes human chondrosarcoma migration through activation of intercellular adhesion molecule-1 [23], which implies that WISP-3 mediates metastasis in chondrosarcoma. However, it remains unclear as to whether WISP-3 promotes VEGF-A production to enhance tumor-regulated angiogenesis in human chondrosarcoma. In this current report, we studied the role of WISP-3 in VEGF-A-regulated angiogenesis and examined the regulation of miRNA in human chondrosarcoma cells.

## RESULTS

### WISP-3 induces VEGF-A-mediated angiogenesis

**Figure 1:**
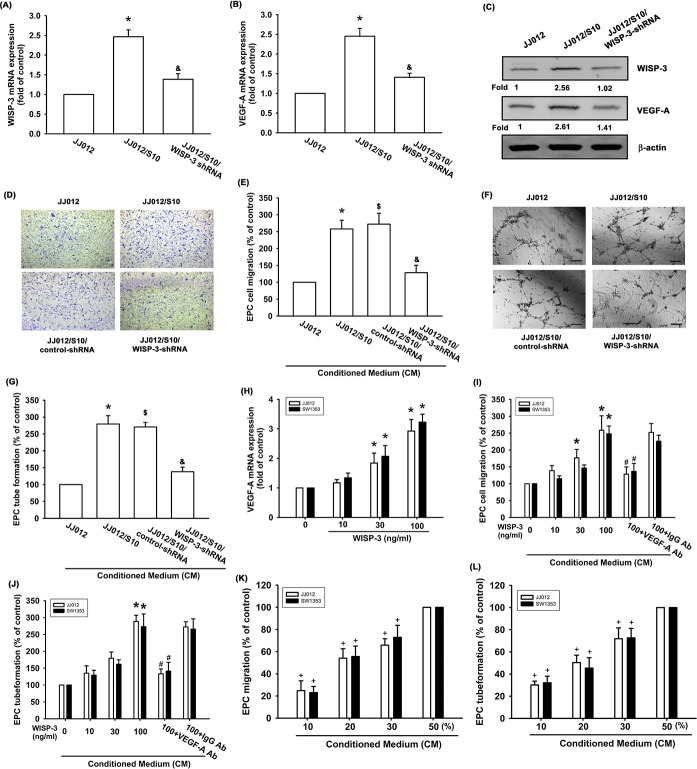
WISP-3 promotes VEGF-A production and angiogenesis in human chondrosarcoma WISP-3 and VEGF-A expression in indicated cells were examined by qPCR (n=5) (**A&B**) and Western blotting (n=3) (**C**). The CM was collected from indicated cells (JJ012, JJ012/S10, JJ012/S10/control-shRNA or JJ012/WISP-3-shRNA cells) then applied to EPCs. The cell migration and tube formation were examined by Transwell (n=4) (**D&E**) and tube formation assay (n=4) (**F&G**). (**H**) JJ012 cells were incubated with various concentrations of WISP-3 (10–100 ng/mL) for 24 h, and VEGF-A expression was examined by qPCR (n=5). (**I&J**) Chondrosarcoma cells were incubated with various concentrations of WISP-3 for 24 h or incubated with WISP-3 (100 ng/mL) for 24 h followed by stimulation with VEGF-A or IgG antibody (5 mg/mL) for 30 min. The medium was collected as CM and then applied to EPCs. The cell migration and tube formation were examined by Transwell **(K)** and tube formation assay (n=4) (**L**). EPCs cells were incubated with indicated osteosarcoma CM (10-50%) for 24 h. The cell migration and capillary-like structure formation were examined by Transwell and tube formation assays (n=4). Scale bar = 200 μm. Quantitative results are expressed as the mean ± SEM. *P < 0.05 as compared with the control group; ^#^*P* < 0.05 as compared with the WISP-3-treated group; ^&^*P* < 0.05 as compared with the JJ012/S10/control-shRNA group; ^$^*P* < 0.05 as compared with the JJ012/S10/control-shRNA group.^+^*P* < 0.05 as compared with the 50% CM group.

We have previously reported that WISP-3 enhances tumor metastasis in human chondrosarcoma cells [23]. In addition, we previous established highly migratory JJ012(S10) cells by using Transwell [24]. Here we revealed that JJ012(S10) cells show higher expression of WISP-3 and VEGF-A expression as compared with JJ012 cells (Figure 1A-1C). Knockdown of WISP-3 reduced WISP-3 and VEGF-A expression (Figure 1A-1C). The effects of WISP-3-mediated chondrosarcoma cell angiogenesis were evaluated on EPC migration and tube formation [25]. CM from highly migratory JJ012(S10) cells enhanced migration and tube formation in EPCs (Figure 1D-1G). However, CM collected from JJ012/S10/WISP-3-shRNA cells abolished migration and tube formation in EPCs (Figure 1D-1G). Direct application of WISP-3 in JJ012 cells enhanced VEGF-A expression as well as migration and tube formation in EPCs (Figure 1H-1J). In addition, similar effects were found in other chondrosarcoma cell lines (SW1353 cells) (Figure 1H-1J). VEGF-A mAb, but not control IgG, abolished the effects of WISP-3 on VEGF-A-induced EPC migration and tube formation (Figures 1I and 1J), which suggests that WISP-3 induces angiogenesis in a VEGF-A-dependent manner. In addition, chondrosarcoma CM promoted EPC migration and tube formation in a concentration-dependent manner (Figures 1K and 1L). These data indicate that WISP-3 increases VEGF-A production and promotes angiogenesis in human chondrosarcoma cells.

### WISP-3 induces VEGF-A expression and angiogenesis via the c-Src/p38 pathway

**Figure 2:**
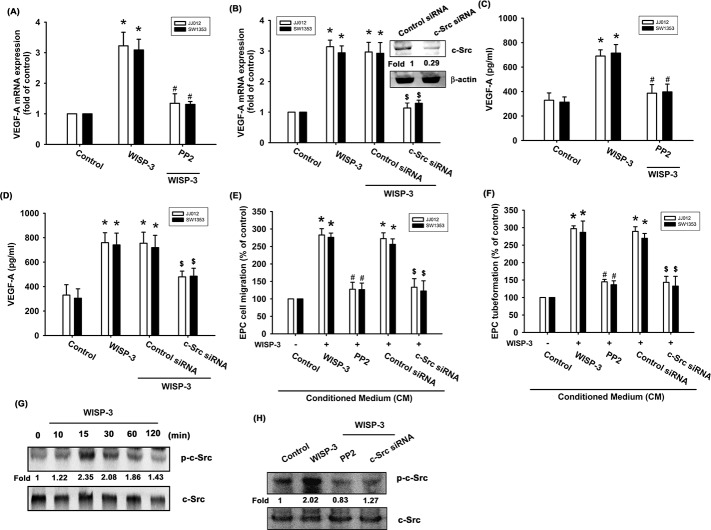
The c-Src pathway is mediated in WISP-3-induced VEGF-A production and angiogenesis (**A-D**) Cells were pretreated with c-Src inhibitor (PP2; 3 μM) for 30 min or pretransfected with c-Src siRNA for 24 h, and then stimulated with WISP-3 for 24 h; VEGF-A expression was measured by qPCR (n=5) and ELISA (n=4). The CM was applied to EPCs and analyzed for migration activity (n=4) (**E**) as well as tube formation activity (n=4) (**F**). (**G**) JJ012 cells stimulated with WISP-3 for the indicated times were analyzed by Western blotting with an antibody against c-Src (n=3). (**H**) JJ012 cells pretreated with pharmacologic inhibitor and siRNA as indicated were then incubated with WISP-3 for 30 min and analyzed by Western blotting with the c-Src antibody (n=3). Quantitative results are expressed as the mean ± SEM. *P < 0.05 as compared with the control group; ^#^*P* < 0.05 as compared with the WISP-3-treated group; ^$^*P* < 0.05 as compared with the control siRNA group.

It was been suggested that the c-Src signaling pathway is involved in cancer progression steps, for example metastasis and angiogenesis [26]. Incubation with a c-Src inhibitor (PP2) for 30 min or transfection with a siRNA for 24 h diminished WISP-3-promoted VEGF-A expression (Figure 2A-2D). These agents also abolished WISP-3-enhanced tube formation and migration in EPCs (Figure 2E and 2F). Stimulation with WISP-3 increased c-Src phosphorylation (Figure 2G). Pretreatment of cells with c-Src inhibitor or siRNA abolished WISP-3-boosted c-Src phosphorylation (Figure 2H). These data imply that WISP-3 induces VEGF-A expression and angiogenesis in chondrosarcomas via the c-Src pathway.

**Figure 3:**
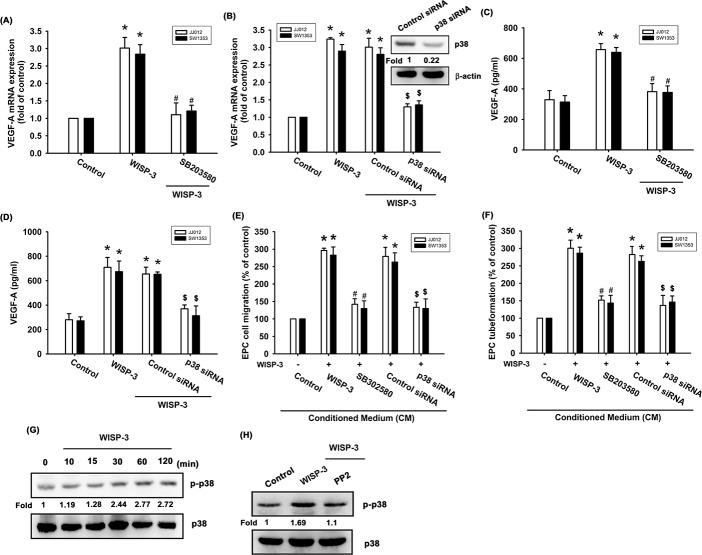
p38 activation is mediated in WISP-3-induced VEGF-A production and angiogenesis (**A-D**) Cells were pretreated with p38 inhibitor (SB203580; 10 μM) for 30 min or pretransfected with siRNA for 24 h then stimulated with WISP-3 for 24 h; VEGF-A expression was measured by qPCR (n=5) and ELISA (n=4). The CM was applied to EPCs and analyzed for migration activity (n=4) (**E**) as well as tube formation activity (n=4) (**F**). JJ012 cells treated with WISP-3 for the indicated times were analyzed by Western blotting with a p38 antibody (n=3) (**G**). JJ012 cells pretreated with pharmacologic inhibitor as indicated were then incubated with WISP-3 for 120 min and analyzed by Western blotting with p38 antibody (n=3) (**H**). Quantitative results are expressed as the mean ± SEM. *P < 0.05 as compared with the control group; ^#^*P* < 0.05 as compared with the WISP-3-treated group; ^$^*P* < 0.05 as compared with the control siRNA group.

p38 is a downstream molecule in c-Src signaling [27, 28]. We therefore examined whether WISP-3 stimulates the p38 signaling pathway. We found that pretreatment with a p38 inhibitor (SB203580) for 30 min or pretransfected with p38 siRNA for 24 h abolished WISP-3-induced increased VEGF-A expression as well as EPC tube formation and migration (Figure 3A-3F). The pharmacologic inhibitors (PP2 and SB203580) did not affect basal VEGF-A expression, EPC migration or tube formation (Supplementary Figure 1). p38 phosphorylation was increased after WISP-3 treatment (Figure 3G). Incubation with c-Src inhibitor antagonized WISP-3-induced p38 phosphorylation (Figure 3H), suggesting that WISP-3 induces VEGF-A expression in chondrosarcomas and promotes EPCs angiogenesis through the c-Src and p38 pathways.

### WISP-3 increases VEGF-A expression and angiogenesis by inhibiting miR-452

**Figure 4:**
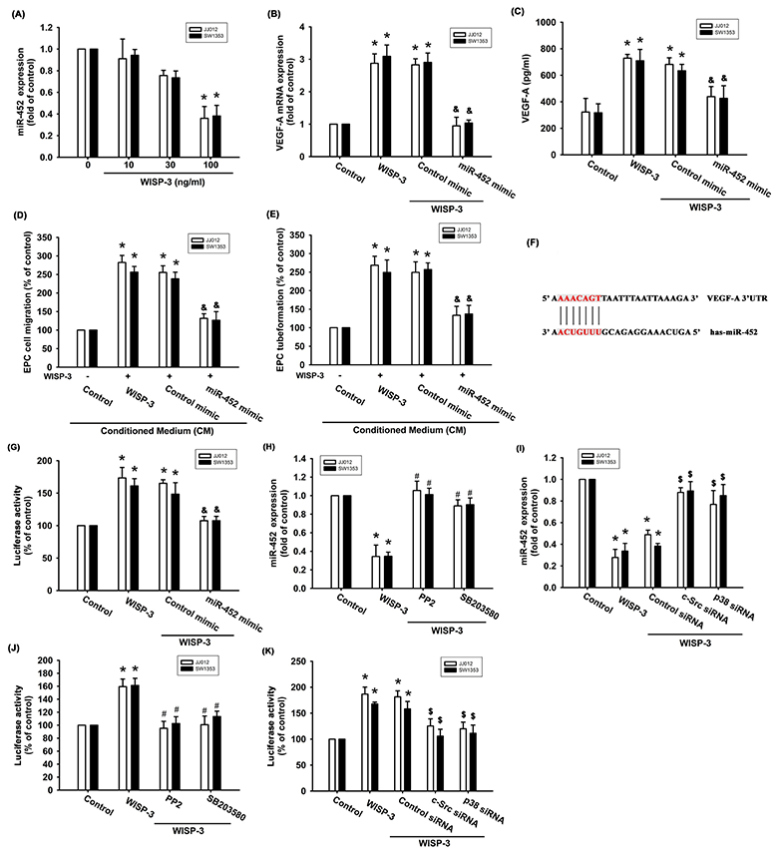
WISP-3 promotes VEGF-A and angiogenesis via inhibition of miR-452 (**A**) Cells were incubated with WISP-3 for 24 h and the miR-452 was examined by qPCR (n=5). Cells were pretransfected with the miRNA as indicated then incubated with WISP-3 for 24 h; VEGF-A expression was measured by qPCR (n=5) and ELISA (n=4) (**B&C**). The CM was applied to EPCs and analyzed for migration activity (n=4) (**D**) as well as tube formation activity (n=4) (**E**). Cells were pretransfected with miRNA as indicated then stimulated with WISP-3 for 24 h. The VEGF-A promoter luciferase was analyzed (n=4) (**F&G**). Cells pretreated with pharmacologic inhibitors or pretransfected with siRNAs as indicated were then stimulated with WISP-3; miR-452 expression (n=4) (**H&I**) and VEGF-A promoter luciferase (n=5) (**J&K**) were analyzed. Quantitative results are expressed as the mean ± SEM. *P < 0.05 as compared with the control group; ^#^*P* < 0.05 as compared with the WISP-3-treated group; ^&^*P* < 0.05 as compared with the control mimic group; ^$^*P* < 0.05 as compared with the control siRNA group.

Increasing evidence has suggested that miRNAs are critical regulators of VEGF-A production and angiogenesis during cancer progression [15, 16]. Use of open-source software (PicTar, miRDB, and TargetScan) to predict and identify target miRNAs in this study found that the 3′UTR region of VEGF-A mRNA harbors potential binding sites for 13 candidate miRNAs, and that the greatest downregulation of miR-452 occurs after WISP-3 stimulation (Supplementary Figure 2). Exogenous WISP-3 inhibited miR-452 expression in a concentration-dependent manner (Figure 4A). To investigate whether miR-452 regulated WISP-3-induced VEGF-A expression and angiogenesis, the miR-452 mimic was used. miR-452 mimic transfection diminished WISP-3-enhanced VEGF-A expression (Figures 4B and 4C). Conversely, the miR-452 mimic also inhibited WISP-3-boosted EPC migration and tube formation (Figure 4D and 4E). We then examined whether miR-452 controls the 3′UTR region of VEGF-A (Figure 4F). We found that WISP-3 promoted VEGFA-3′UTR luciferase activity (Figure 4G). Incubation with c-Src and p38 inhibitors or siRNAs reversed WISP-3-mediated miR-452 expression and VEGFA-3′UTR luciferase activity (Figure 4H-4K), indicating that miR-452 inhibits VEGF-A protein production through integration with the 3′UTR region of the human VEGF-A gene via the c-Src and p38 pathways.

### Inhibiting WISP-3 expression suppresses tumor-induced angiogenesis *in vivo*

**Figure 5:**
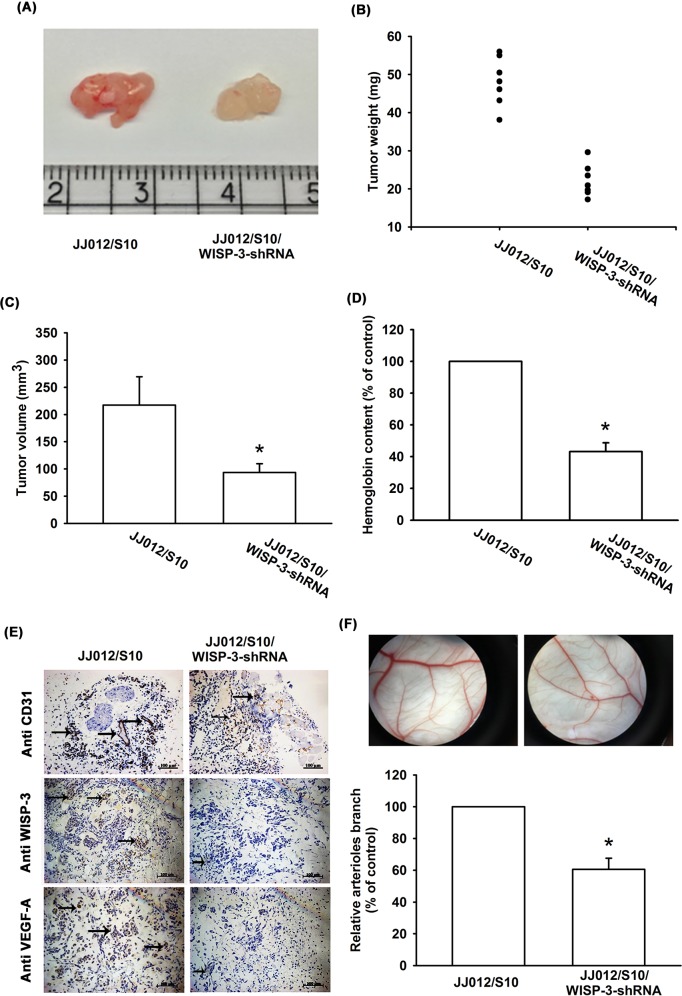
WISP-3 knockdown decreases tumor-induced angiogenesis *in vivo* At 28 days after mice were injected, tumor size and weight were measured (n=7) (**A-C**), and quantified for hemoglobin content (**D**), as well as embedded in paraffin, and sections were immunostained using CD31, WISP-3, and VEGF-A antibody (**E**). (**F**) Chick embryos were incubated for 4 days with CM from JJ012/S10 or JJ012/S10/WISP-3-shRNA cells and then resected, fixed, and photographed with a stereomicroscope (n=5). Quantitative results are expressed as the mean ± SEM. *P < 0.05 as compared with the JJ012/S10 group.

We also examined whether WISP-3 regulates angiogenesis *in vivo*. In the tumor-induced angiogenesis model, we found that knockdown WISP-3 inhibited tumor growth in SCID mice (Figure 5A-5C). In addition, inhibiting WISP-3 expression reduced chondrosarcoma-promoted angiogenesis *in vivo* (by analysis of the hemoglobin content of the tumor) (Figure 5D). Knockdown of WISP-3 expression reduced the expression of the vessel markers CD31, WISP-3, and VEGF-A on immunohistochemistry (Figure 5E). WISP-3-regulated angiogenesis was then examined by the *in vivo* CAM assay. The data showed that CM from the JJ012/S10 group promoted vessel formation (Figure 5F). However, CM from JJ012/S10/WISP-3-shRNA group decreased vessel formation in the CAM model (Figure 5F). These results indicate that WISP-3 enhances angiogenesis and tumor growth *in vivo*.

**Figure 6:**
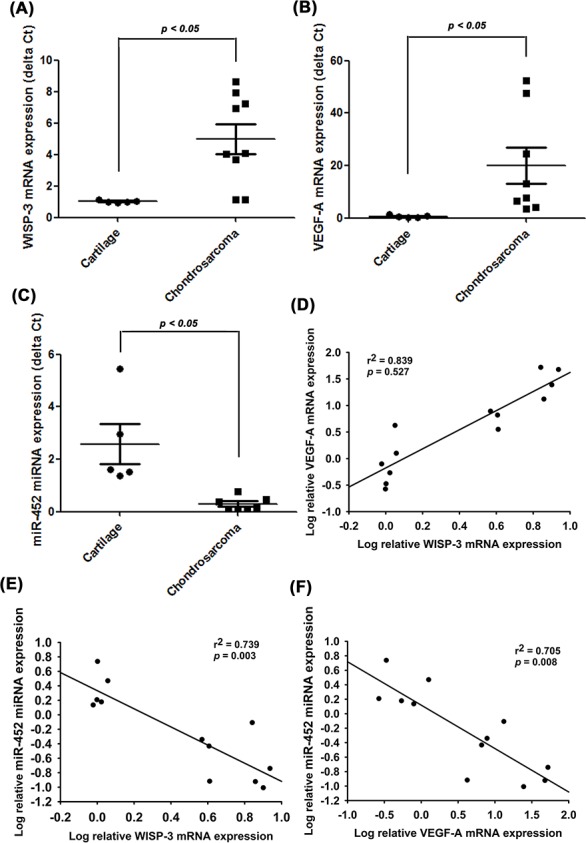
WISP-3, VEGF-A and miR-452 expression have important clinical significance in patients with chondrosarcoma mRNA expression of WISP-3, VEGF-A, and miR-452 in normal cartilage (n=5) and chondrosarcoma tissue (n=8) were examined by qPCR (**A-C**). The correlations between WISP-3/VEGF-A (**D**), WISP-3/miR-452 (**E**) and VEGF-A/miR-452 (**F**). Quantitative results are expressed as the mean ± SEM. *P < 0.05 as compared with the cartilage group.

To further examine the clinical significance of WISP-3 in chondrosarcoma angiogenesis, we analyzed the expression profiles of WISP-3 and VEGF-A in chondrosarcoma patients. Both profiles were higher in chondrosarcoma specimens than in normal cartilage (Figure 6A and 6B); lower expression of miR-452 was seen in chondrosarcoma patients (Figure 6C). These results reveal a positive correlation between WISP-3 mRNA expression and VEGF-A (Figure 6D), while miR-452 expression was negatively correlated with WISP-3 and VEGF-A expression (Figure 6E and 6F).

## DISCUSSION

While chondrosarcoma is relatively rare, it is notoriously aggressive and has high metastatic potential and a poor prognosis [29]. Angiogenesis is a key step in cancer metastasis, promoting cancer progression via synthesis of new blood vessels [30]. Increasing evidence indicates that enhanced expression of VEGF-A increases tumor relapse and poor prognosis. Therefore, VEGF-A represents a potential target for preventing angiogenesis and metastasis [31]. This study describes the clinical significance of WISP-3 and VEGF-A in patients with chondrosarcoma. We also found that WISP-3 promotes the production and secretion of the angiogenic factor VEGF-A through the inhibition of miR-452 via the c-Src and p38 pathways in human chondrosarcoma cells, subsequently inducing angiogenesis in human EPCs, suggesting that WISP-3 and miR-452 may be novel molecular targets for inhibiting VEGF-A-induced angiogenesis in chondrosarcoma.

The CCN family of proteins has been indicated to enhance tumor proliferation, migration, and metastasis. Nevertheless, it has the opposite effect in some tumors. For example, the level of connective tissue growth factor (CTGF/CCN2) in gastric, pancreatic, and breast cancer is correlated to worse prognosis; however, the opposite effect is seen in ovarian, lung and colorectal cancer [32]. Similarly, WISP-3 has been associated with both tumor progression and suppression. In gastric cancer, knockdown WISP-3 reduced cell proliferation and migration [33]. We previous reported WISP-3 promoted chondrosarcoma migration through upregulated ICAM-1 production [23]. Overexpression of WISP-3 in breast cancer suppressed cell metastasis and growth [34], suggesting WISP-3 is a critical tumor suppressor in breast cancer. These results indicate WISP-3 plays an oncogene or tumor suppressor role in different tumor cells.

Increasing reports suggest that c-Src, a potential candidate signaling pathway, regulates cancer metastasis and angiogenesis [35, 36]. In the current study, we report that an c-Src inhibitor or siRNA diminished WISP-3-induced production of VEGF-A. Conversely, these agents diminished WISP-3-induced EPC migration and tube formation. Stimulation of chondrosarcoma cells with WISP-3 enhanced phosphorylation of c-Src, indicating that activation of c-Src plays a key role in WISP-3-induced VEGF-A expression and angiogenesis. Activation of p38 is a critical downstream signaling of the c-Src pathway [37]. In addition, the c-Src inhibitor reduced WISP-3-enhanced p38 phosphorylation. These findings imply that the c-Src-dependent p38 pathway plays a critical role, even in WISP-3-promoted VEGF-A production and angiogenesis.

miRNAs have been widely implicated as being capable of reducing biogenesis in human cancer cells [16]. Increasing evidence suggests that several miRNAs can reduce tumor progression via direct repression of VEGF-A. miR-199a, miR-200b, miR-206, miR-210, and miR-374b have been shown to inhibit metastasis and angiogenesis in several human cancer cells by targeting VEGF-A [18, 25, 38, 39]. This current study has indicated that WISP-3 markedly inhibits the expression of miR-452 in human chondrosarcoma cells *in vitro* and *in vivo*. miR-452 mimic transfection reduced WISP-3-promoted VEGF-A production and EPC migration and tube formation. We also found that miR-452 directly reduced VEGF-A production via integration with the 3′UTR of the human VEGF-A gene, negatively modulating VEGF-A-induced angiogenesis. These results provide insight into the potential miRNA-based molecular diagnosis and treatment for VEGF-A-regulated cancer angiogenesis.

The rate-limiting step in metastasis is the acquisition of motility by a tumor cell, which is a critical stage in cancer progression. In chondrosarcoma, lung metastasis is the major cause of mortality. Discovery of an antiangiogenic, antimetastatic therapy could potentially provide enormous benefits in chondrosarcoma patients. In this current study, we found that WISP-3 induced VEGF-A expression and subsequently promoted angiogenesis and tumor growth in human chondrosarcoma cells via suppressing miR-452 through the c-Src and p38 signaling cascades. These findings may provide a better understanding of the mechanisms of angiogenesis and may lead to the development of effective therapies for chondrosarcoma.

## MATERIALS AND METHODS

### Materials

Anti-mouse and anti-rabbit IgG-conjugated horseradish peroxidase as well as rabbit monoclonal antibodies (mAbs) specific for WISP-3, p38, p-p38 (Tyr^182^), c-Src, and β-actin were obtained from Santa Cruz Biotechnology (Santa Cruz, CA, USA). Rabbit polyclonal antibody specific for p-Src (Tyr^527^) was purchased from Cell Signaling and Neuroscience (Danvers, MA, USA). A VEGF-A ELISA kit was obtained from PerpoTech (Rocky Hill, NJ, USA). Control IgG and rabbit mAb specific for VEGF-A were obtained from Abcam (Cambridge, MA, USA). Control miRNA, miR-452 mimic, MMLV RT kit, Trizol and Lipofectamine 2000 were purchased from Invitrogen (Carlsbad, CA, USA). The TaqMan MicroRNA Reverse Transcription kit and the TaqMan assay kit were obtained from Thermo Fisher Scientific (Grand Island, NY, USA). c-Src, p38 and control siRNA were purchased from Dharmacon Research (Lafayette, CO, USA). PP2 and SB203580 were obtained from Sigma-Aldrich (St. Louis, MO, USA).

### Cell culture

The human chondrosarcoma cell line (JJ012) was obtained from Dr. Sean P. Scully (University of Miami School of Medicine, Miami, FL). JJ012 cells were cultured in Dulbecco's modified Eagle's medium (DMEM)/α-MEM supplemented with 10% fetal bovine serum (FBS), and maintained at 37°C in a humidified atmosphere of 5% CO_2_. The human chondrosarcoma cell line (SW1353) was obtained from the American Type Culture Collection. Cells were maintained in humidified air containing 5% CO_2_ at 37°C with Dulbecco's modified Eagle's medium (DMEM), 10% FBS, 100 units/ml penicillin and 100 mg/ml streptomycin (Gibco-BRL Life technologies; Grand Island, NY, USA).

Endothelial progenitor cells (EPCs) were prepared as described previously [40, 41]. EPCs were cultured in MV2 complete medium that contained MV2 basal medium and growth supplement (PromoCell, Heidelberg, Germany), as well as 20% defined FBS (HyClone, Logan, UT, USA). Cultures were seeded on 1% gelatin-coated plastic ware and maintained at 37°C in a humidified 5% CO_2_ atmosphere.

The highly migratory JJ012(S10) cells were selected in our laboratory [24]. Subpopulations of JJ012 cells were selected according to their differential migration ability by using Transwell. After overnight migration, cells that had penetrated through pores and migrated to the underside of filters were trypsinized and harvested for a second round of selection. After 10 rounds of selection, a migration-prone subline was designated as JJ012 (S10). The original cells were designated as JJ012 (S0).

For generation of stable cell lines, the JJ012/S10 cells were seeded on plates and infected with WISP-3-shRNA (Santa Cruz, CA, USA; sc-39339) or control-shRNA (Santa Cruz, CA, USA; sc-108060) by prepared lentivirus. Stable transfectants were selected with puromycin (10 mg/mL) after 24 h transfection. The selection medium was then replaced every 2 days. The resistant clones were established after 2 weeks of selection.

### Collection of clinical samples

The study protocol was approved by the Institutional Review Board of China Medical University Hospital. All patients gave written consent before enrollment. Tumor tissue specimens were collected from patients diagnosed with chondrosarcoma who underwent surgical resection at China Medical University Hospital.

### Collection of conditioned medium and ELISA assay

Human chondrosarcoma cells were plated in 6-well dishes and grown to confluence. The culture medium was then exchanged with serum-free DMEM/α-MEM medium. Cells were pretreated for 30 min with pharmacological inhibitors or pretransfected for 24 h with siRNAs followed by treatment with WISP-3 for 24 h. The medium was collected as conditioned medium (CM), and stored at −20°C until use. The secreted VEGF-A expression was then examined by VEGF-A ELISA assay kit according to the manufacturer's instructions.

### EPCs tube formation assay

EPCs were cultured at a density of 3 × 10^4^ (50% MV2 medium and 50% chondrosarcoma cell CM) and applied to the 48-well plates, which were precoated with 150 μL Matrigel. EPC tube formation was photographed after 6 h and the number of tube branches was counted.

### EPCs migration assay

This process was performed with Transwell inserts (8-μm pore size; Costar, NY, USA). Chondrosarcoma cells were pretreated with designated inhibitors or vehicle (0.1% dimethyl sulfoxide [DMSO]) or pretransfected with designated siRNAs, and following stimulation with WISP-3 for 24 h, the CM was collected. The lower chamber contained 150 ml of MV2 complete medium and 150 ml of CM. EPCs (approximately 1 × 10^4^ cells in 200 ml of medium with 10% FBS MV2) were seeded into the upper chamber of a Transwell assay. After 24 h of migration, cells were fixed and stained with 0.05% crystal violet. Migrated cells on the underside of the lower chamber filters were examined and counted under a microscope.

### Western blotting

Cell lysates were prepared as per the method mentioned in our previous study [42, 43]. Proteins were resolved on SDS-polyacrylamide gel electrophoresis and then transferred to polyvinyldifluoride membranes. The blot membranes were blocked with 4% non-fat milk for 1 h at room temperature, and then incubated overnight with primary antibodies at 4°C. The blots were incubated with anti-rabbit or anti-mouse HRP-conjugated secondary antibodies for 1 h at room temperature. Finally, the blots were visualized by the Fujifilm LAS-3000 chemiluminescence detection system (Fujifilm; Tokyo, Japan).

### Quantitative real-time polymerase chain reaction (qPCR)

Total RNA was extracted from chondrosarcoma cells using TRIzol reagent. mRNA was reversely transcribed to complementary DNA using the MMLV RT kit, and qPCR was performed using the TaqMan assay kit. qPCR analysis of miRNAs expression was performed on the StepOnePlus sequence detection system, using the TaqMan MicroRNA Reverse Transcription Kit and normalized to U6 expression.

### Small interfering RNA (siRNA) transfection

ON-TARGETplus siRNAs of c-Src (L00311000), p38 (L00351200), and control (D0018101005) were purchased from Dharmacon Research (Lafayette, CO, USA). Transient transfection of siRNAs was carried out using DharmaFECT1 transfection reagent. The siRNA (100 nM) was formulated with DharmaFECT1 transfection reagent according to the manufacturer's instructions.

### Plasmid construction and luciferase reporter assay

VEGF-A-3′-UTR was constructed into the pmirGLO-Control vector between PmeI and XhoI cutting sites, according to the manufacturer's instructions. The wild-type VEGF-A-3′-UTR was amplified using PCR with primers (5′-AGGGTTTCGGGAACCAGAT-3′ and 5′-CTGGCCTTGCACATTCCT-3′). To analysis 3′-UTR luciferase activity, chondrosarcoma cells were transfected with VEGF-A-3′UTR luciferase plasmid. Cells were lysated after 24 h of transfection, harvested and detected using a luciferase assay system (Promega; Madison, WI, USA).

### Chick chorioallantoic membrane (CAM) assay

Fertilized chicken eggs were incubated at 38°C with 80% humidity. A small window was made in the shell on day 3, and the window was resealed with adhesive tape. On day 7, CM from JJ012/S10 or JJ012/S10/WISP-3-shRNA cells (2 × 10^4^ cells) deposited in the center of the CAM. At 11 days, CAMs were collected for microscopy and photographic documentation. Angiogenesis was quantified by counting the number of blood vessel branches.

### *In vivo* tumor xenograft study

Exponentially growing cultures resuspended in 200 μl of containing 50% serum-free DMEM/α-MEM and 50% Matrigel containing 2 × 10^6^ cells (JJ012/S10/control-shRNA or JJ012/S10/WISP-3-shRNA cells) were transplanted subcutaneously into the right flanks of BALB/c-nu mice (4-week-old males). After 4 weeks the tumor was removed and fixed in 10% formalin, then measured for tumor volume and weight. Hemoglobin content was examined using Drabkin's reagent. All animal work was done in accordance with a protocol approved by the China Medical University (Taichung, Taiwan) Institutional Animal Care and Use Committees.

### Statistics

All quantified results are presented as the means ± SEM of at least three independent experiments. The Student's *t*-test was used to determine statistical differences between two groups. One-way ANOVA with Bonferroni's post-hoc tests were used for statistical comparisons of more than two groups. *p* < 0.05 was considered to be statistically significant.

## SUPPLEMENTARY FIGURES


