# Supplementary material for: WISP-3 inhibition of miR-452 promotes VEGF-A expression in chondrosarcoma cells and induces endothelial progenitor cells angiogenesis
Source: Oncotarget. 2017 Apr 17;8(24):39571–81. doi: 10.18632/oncotarget.17142 (PMC5503633; doi:10.18632/oncotarget.17142)
Supplement: Supplementary file 1 [file oncotarget-08-39571-s001.pdf]

WISP-3 inhibition of miR-452 promotes VEGF-A expression in chondrosarcoma cells and induces endothelial progenitor cells angiogenesis

SUPPLEMENTARY FIGURES

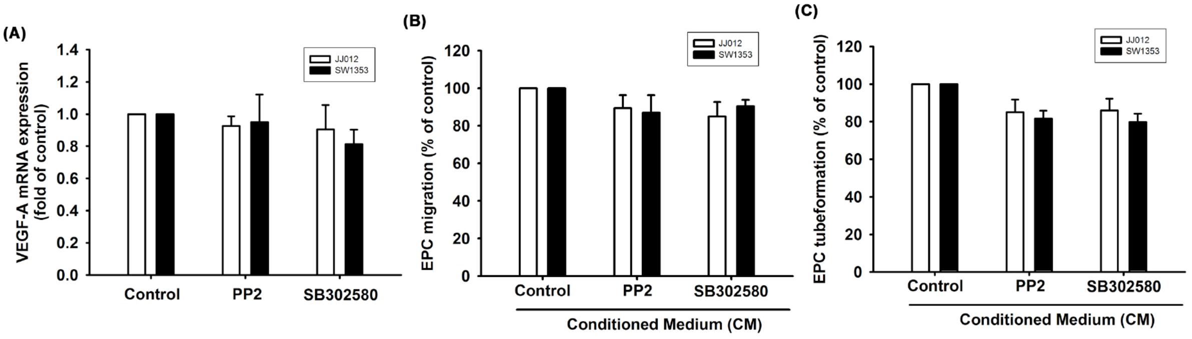

Supplementary Figure 1: The pharmacological inhibitors (PP2 and SB203580) did not affect basal VEGF-A expression, EPCs migration and tube formation. (**A**) Chondrosarcoma cells were treated with 3 μM of PP2 or 10 μM of SB203580 for 24 h, and the VEGF-A expression were measured by the qPCR. The CM was applied to EPCs and analyzed for migration activity (**B**) as well as tube formation activity (**C**). Quantitative results are expressed as the mean ± SEM.

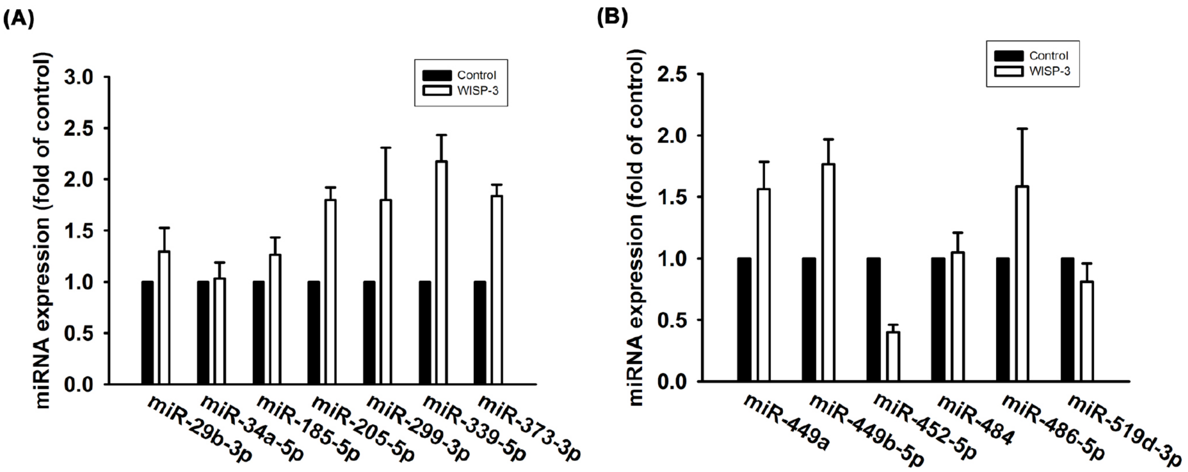

Supplementry Figure 2: WISP-3 affects miRNAs expression in chondrosarcoma cells. Cells were incubated with WISP-3 for 24 h and the miRNA expression was examined by qPCR. Quantitative results are expressed as the mean ± SEM.
